# Substance Abuse and Mental Health: Exploring the Effects on Pediatric Trauma in the COVID-19 Pandemic Era

**DOI:** 10.7759/cureus.95091

**Published:** 2025-10-21

**Authors:** Kylie Scallon, Jessica M Lee, Angela Hanna, Patrick B Thomas, Junghyae Lee, Christopher Oh

**Affiliations:** 1 Trauma, Children's Nebraska, Omaha, USA; 2 Pediatric Surgery, Children's Nebraska, Omaha, USA; 3 Biostatistics, University of Nebraska Medical Center, Omaha, USA; 4 Biology, Washington University, St. Louis, USA

**Keywords:** children, covid-19, drug and alcohol abuse, mental health, pediatric trauma, substance abuse, trauma pediatric

## Abstract

Background

Substance abuse and mental health diagnoses among pediatric patients with trauma have been rising. The COVID-19 pandemic introduced social isolation and stressors that may have increased risky behaviors and worsened mental health in youth.

Objective

The overall objective was to evaluate changes in the proportion of pediatric patients with trauma with mental health and/or substance abuse comorbidities before, during, and after the COVID-19 pandemic.

Methods

A retrospective, single-center cohort study was conducted using trauma registry data (2018-2023) for patients meeting National Trauma Data Standard criteria with documented mental health or substance abuse disorders. Data were analyzed across three periods: pre-pandemic (2018-2019), pandemic (2020-2021), and post-pandemic (2022-2023). Variables included demographics, injury mechanism and severity, toxicology results, admission status, and hospital course.

Results

The proportion of patients with comorbidities increased from 1.11% pre-pandemic to 4.99% during and 8.99% post-pandemic. Most were white, non-Hispanic male patients, with blunt trauma as the predominant mechanism. The patients during the pandemic period had the highest mean injury severity score (6.0), the longest ICU stays (4.3 days), the longest ventilator use (5.0 days), and the greatest need for surgery (50.6%).

Conclusion

The proportion of pediatric patients with trauma and mental health or substance abuse disorders rose nearly nine-fold during and after the pandemic, indicating a lasting impact of pandemic-related disruptions. These patients demonstrated greater injury severity and hospital resource use, underscoring the need for continued mental health screening and intervention in pediatric trauma care.

## Introduction

The prevalence of substance abuse and mental health diagnoses in pediatric populations has been increasing steadily [1]. The Coronavirus disease 2019 (COVID-19) pandemic brought unprecedented attention to mental health diagnoses, including anxiety, major depressive disorder (MDD), attention deficit hyperactivity disorder (ADHD), and mood disorders. Adolescents experienced a marked increase in these conditions due to pandemic-related stressors, including social isolation, masking requirements, daycare and school closures, fear of infection, grief from losing loved ones, and uncertainty about the future [2]. While measures to control COVID-19 transmission were essential, they inadvertently led to decreased physical activity, fewer primary care visits, and increased mental health needs [2].

Children and adolescents faced increased risk of poor social and emotional outcomes due to home confinement, cancelled activities, and separation from classmates, friends, and teachers [2]. However, some studies have shown an increase in outdoor injuries, suggesting that children were not consistently confined to home. In Nebraska specifically, the absence of a “stay-at-home” mandate, combined with school closures, may have resulted in reduced adult supervision [3]. Schools traditionally play a vital role in monitoring student well-being, addressing nutritional needs, and providing physical and mental health support [4]. Bessoff et al. found an overall decrease in pediatric trauma volume in communities with shelter-in-place mandates; however, injury severity was higher among those patients compared to control populations [5].

The most recent comprehensive data published on mental health in America was collected through 2022 and has a disclaimer stating the COVID-19 pandemic significantly impacted the data collection process, and therefore cannot accurately be compared to previous years [1]. Nevertheless, these data revealed that 20% of United States youth aged 12-17 reported experiencing severe major depression in 2022. Additionally, 9% of youth in the United States reported one or multiple substance use disorders [1]. Other studies have suggested rising anxiety and depression levels among children impacted by the pandemic [6,7]. Among injured patients, mental health disorders are more prevalent, and the rate of substance use disorder is higher compared to the general population [8,9]. Adolescence is the stage of life when first exposure to drugs and alcohol most often occurs, even without the added impact of a pandemic. Substance use during this period increases the likelihood of high-risk behaviors and is closely linked to a higher incidence of traumatic injuries in this population [10].

Although numerous studies have examined the psychological impact of the pandemic on children and adolescents, there is limited research evaluating how these mental health and substance use trends intersect with pediatric populations that have undergone trauma. Most existing literature focuses on general mental health outcomes or overall trauma volume rather than comorbid psychiatric or substance use disorders among injured pediatric patients. Furthermore, regional differences, such as the absence of a stay-at-home mandate in Nebraska, may have uniquely influenced trauma mechanisms and comorbidity patterns, yet these factors remain poorly characterized.

Based on these concerning trends, we hypothesized that our hospital would see an increase in comorbid mental health or substance abuse disorders among patients receiving care for injuries during and after the COVID-19 pandemic compared to pre-pandemic periods.

## Materials and methods

Study design

This is a single-center, retrospective cohort study. This study was reviewed and approved by the Institutional Review Board of the University of Nebraska Medical Center (approval no. 0148-24-EP).

Population and setting

We evaluated patients at a free-standing American College of Surgeons verified Level II pediatric trauma center (Children’s Nebraska in Omaha, Nebraska, US) with a total emergency department (ED) annual volume of 38,000 patients. Located in the Midwest, it is the only free-standing pediatric hospital in the state. Approximately 800 patients are added to the trauma registry each year. Patients aged 0-17 were screened for inclusion.

Data collection

This study included all pediatric patients entered in our trauma registry from January 2018 through December 2023. To be included in the trauma registry, the patient must have been admitted to our trauma center or transferred from an outside hospital. Specific aims of this study were to compare patients presenting to the hospital with comorbid mental health or substance abuse disorders two years prior to COVID as control (2018-2019), the two years during the pandemic (2020-2021), and the two years post-pandemic (2022-2023). Variables analyzed included age, gender, gender identity, race, ethnicity, injury location, post ED disposition, use of protective devices (seatbelts, helmets, etc.), injury mechanism, trauma activation level, Glasgow Coma Score (GCS) [11] on admission, alcohol screening completed, ethanol level on admission, drug screen performed, procedures performed, diagnosis, injury severity score (ISS) [12], total intensive care unit (ICU) days, total ventilator days, and hospital discharge disposition.

Alcohol screening guidelines were the same throughout these periods, though it modified slightly to meet required changes. The Car, Relax, Alone, Forget, Family/Friends, Trouble (CRAFFT) screening [13] was performed on all trauma patients aged 12 or older that were admitted to the hospital for greater than 24 hours. However, in 2022, the American College of Surgeons (ACS) changed alcohol screening requirements to all admitted trauma patients greater than 12 years old, regardless of length of admission [14]. The CRAFFT tool [13] is free to use and is available online. Inclusion for a mental health or substance use comorbidity was by self-reporting or having a previous diagnosis in the health record.

Statistical analysis

Continuous variables were assessed for normality to determine the appropriateness of parametric versus non-parametric methods. Categorical variables with excessive granularity were recoded into conceptually meaningful categories to enhance interpretability and maintain statistical power. Based on the distributional assessments, comparisons across the three time periods (pre-pandemic, pandemic, and post-pandemic) were conducted using Analysis of Variance (ANOVA) test for continuous outcomes, with statistical significance defined as a two-sided p-value <0.05. Categorical outcomes, including binary variables such as operative status, were initially compared using Chi-square tests. All analyses were conducted using SAS software, version 9.4 (SAS Inc., Cary, NC, US).

## Results

Over the three epochs, the total number of patients and those presenting with comorbid mental health or substance abuse disorders were 1142 and 12 (1.1%) in the pre-pandemic era, 1662 and 83 (5.0%) in the pandemic era, and 1768 and 159 (9.0%) in the post-pandemic era, respectively. The demographic characteristics of the patients with comorbidity were not different across the pre-pandemic, pandemic, and post-pandemic periods regarding age, gender, race, or ethnicity distributions (Table 1).

**Table 1:** Demographic and clinical characteristics across pre-pandemic, pandemic, and post-pandemic periods ^1^Age: F(2, 251)=0.41, p=0.66, η²=0.0033; ^2^ANOVA F-test p-value; ^3^Chi-Square p-value; SD, standard deviation *Ethnicity data were missing for one patient in the post-pandemic era

	Pre-pandemic	Pandemic	Post-pandemic	P-value
No of patients	N=12	N=83	N=159	
Age^1^				0.66391^2^
Mean (SD)	12.3 (2.34)	13.2 (3.72)	13.0 (3.55)	
Gender, n (%)				
Female	2 (16.7%)	16 (19.3%)	47 (29.6%)	
Male	10 (83.3%)	67 (80.7%)	112 (70.4%)	
Race, n (%)				0.66391^3^
Black or African American participants	0 (0.0%)	5 (6.0%)	12 (7.5%)	
White participants	11 (91.7%)	67 (80.7%)	126 (79.2%)	
Participants from other races (Asian, American Indian, and two or more races)	1 (8.3%)	11 (13.3%)	21 (13.2%)	
Hispanic or Latino participants, n (%)*	1 (8.3%)	9 (10.8%)	14 (8.9%)	0.99072^3^

There was no significant difference between the groups with respect to the utilization of protective devices, injury type, initial GCS, post-ED disposition, or ISS. There was an increase in the number of patients activated as either a full or partial trauma over time (Table 2).

**Table 2:** Clinical features, screening, and discharge outcomes across the pre-pandemic, pandemic, and post-pandemic periods ^1^Chi-Square p-value; ^2^ANOVA F-test p-value; ^3^GCS admission: F(2, 251)=1.33, p=0.27, η²=0.0105; ​​​​​​​^4^ISS: F(2, 247)=0.18, p=0.83 ,η²=0.0015; GCS: Glasgow Coma Scale [11]; SD: standard deviation; ED: emergency department; ISS: injury severity score [12]

	Pre-pandemic (N=12)	Pandemic (N=83)	Post-pandemic (N=159)	P-value
Protective device, n (%)				0.0612^1^
Yes	0 (0.0%)	3 (3.6%)	18 (11.5%)	
Injury type, n (%)				0.0422^1^
Blunt	12 (100.0%)	65 (78.3%)	146 (91.8%)	
Drowning or suffocation	0 (0.0%)	2 (2.4%)	3 (1.9%)	
Other and unspecified	0 (0.0%)	8 (9.6%)	3 (1.9%)	
Penetrating	0 (0.0%)	8 (9.6%)	7 (4.4%)	
Trauma response level, n (%)				0.0162^1^
Full	0 (0.0%)	3 (3.6%)	12 (7.5%)	
Partial	4 (33.3%)	35 (42.2%)	92 (57.9%)	
No Trauma Activation	8 (66.7%)	45 (54.2%)	55 (34.6%)	
GCS on admission^3^				0.2673^2^
Mean (SD)	14.0 (3.46)	14.4 (2.59)	14.7 (1.49)	
ED disposition, n (%)				0.2272^1^
Admitted	11 (91.7%)	57 (68.7%)	95 (59.7%)	
Discharged	1 (8.3%)	23 (27.7%)	61 (38.4%)	
Mental health facility	0 (0.0%)	2 (2.4%)	1 (0.6%)	
Transfer to acute care facility	0 (0.0%)	1 (1.2%)	2 (1.3%)	
ISS^4^				0.8323^2^
Mean (SD)	5.6 (4.62)	6.0 (6.98)	5.5 (6.03)	
Drug screen, n (%)				0.0011¹
Yes	0 (0.0%)	9 (10.8%)	23 (14.5%)	
Alcohol screen, n (%)				<0.0001
Yes	0 (0.0%)	14 (16.9%)	82 (51.6%)	
Positive ethanol level				0.6692^1^
Yes		3/14 (21.4%)	2/82 (2.4%)	
Discharge to, n (%)				0.0021^1^
Acute care facility	0 (0.0%)	1 (1.2%)	4 (2.5%)	
Correctional facility	1 (8.3%)	0 (0.0%)	0 (0.0%)	
Home	11 (91.7%)	73 (88.0%)	150 (94.3%)	
Mental health	0 (0.0%)	7 (8.4%)	4 (2.5%)	
Morgue	0 (0.0%)	1 (1.2%)	0 (0.0%)	
Rehab	0 (0.0%)	1 (1.2%)	1 (0.6%)	

The findings indicate a significant increase in both alcohol and drug screenings over time, with a corresponding rise in positive test results (Table 2). Alcohol screening increased from 0.0% in the pre-pandemic period to 16.9% during the pandemic and 51.6% post-pandemic (p<0.00012), identifying a shift in screening practices due to the previously mentioned change in ACS screening requirements. There were three patients during the pandemic and two patients post-pandemic who had positive ethanol levels upon arrival to the emergency department. Drug screening rose from 0.0% pre-pandemic to 10.8% during the pandemic and 14.5% in the post-pandemic periods (p=0.00052).

The ANOVA F-tests showed no statistically significant effects of age, GCS at admission, or ISS. None of these tested predictors showed significant effects on the dependent variable, and all effect sizes were very small to negligible (<1 to 1.1%). 

The highest number of operative procedures were performed during the pandemic period (42, 50.6%), and the likelihood of undergoing a procedure was significantly higher compared to the pre-pandemic period, with odds increasing by 2.3 times (95% CI: 1.33 - 3.97, p=0.003). In the post-pandemic period, procedure rates declined, with the odds being 0.22 times relative to during the pandemic (95% CI: 0.06 - 0.77, p=0.018. When comparing the pre-pandemic and post-pandemic periods, no significant difference in procedure rates was observed (OR=0.51, 95% CI: 0.14 - 1.83, p=0.304).

Most patients were discharged home after their hospital course, however 7/83 (8.4%) patients during the pandemic and 4/159 (2.5%) patients in the post-pandemic period were transferred to a mental health facility. One patient died due to injuries during the pandemic era (Table 2).

## Discussion

Our study identified a significant increase in the frequency of diagnosed mental health disorders or substance abuse disorders among children presenting to the hospital with injuries during and after the COVID-19 pandemic. Several factors may explain these findings. The pandemic likely triggered changes in behavior patterns, potentially leading individuals to initiate drug or alcohol use when they had not previously done so. Additionally, some patients may have had pre-existing mental health conditions, but only required emergency care for injuries sustained during or after the pandemic. The pandemic brought mental health issues to the spotlight. It is possible that due to the heightened awareness, more patients were assessed for and diagnosed with mental health comorbidities, which would explain the increase in comorbidities from before the pandemic. Individuals diagnosed with mental health illnesses face an increased risk of injury and demonstrated higher rates of risk-taking behaviors [15,16]. In Nebraska, youth substance use disorder rates increased from 7.13% in 2020 to 8.86% in 2022, while reports of severe major depression increased from 15.7% to 21.12% during the same period [17,1]. Our findings mirror these trends.

A particularly noteworthy aspect of our results concerns surgical interventions. Despite the widespread suspension of non-emergent surgeries during the pandemic [18], our study revealed that patients with comorbid mental health or substance use disorders were significantly more likely to undergo operative procedures during the pandemic compared to the pre-pandemic period. This finding is especially striking given our institution’s policy of performing only emergent surgeries during this time. However, this observation aligns with other severity indicators in our pandemic population, including higher ISS, extended ICU stays, and increased ventilator days.

These results suggest that patients with mental health and substance use disorders not only presented more frequently during and after the pandemic, but also with more severe injuries requiring more intensive interventions. This pattern raises important questions about the complex correlation between mental health challenges, risk-taking behaviors, and traumatic injuries during periods of societal stress. We did not evaluate for a causal relationship between the comorbidity and need for surgical intervention, as we had increased percentage of patients with the comorbidity post-pandemic, but the surgical interventions decreased during that period. However, the continued rise in these presentations beyond the acute period of the pandemic suggests that the psychological impacts of COVID-19 may have long-lasting effects on vulnerable populations, particularly children and adolescents.

Limitations

This study has several limitations. Because trauma registry data are entered manually, there is a risk of data entry errors or unintentional omission of eligible patients. As a single-center study, the findings may not be generalizable to other institutions; a multi-center approach could improve sample size and provide a more robust validation of these results. Additionally, mental health or substance use diagnoses may have been underreported due to stigma or concerns about disclosure. Changes to screening requirements during the study period may also have affected the number of patients identified. Finally, the lack of long-term follow-up limits the ability to evaluate outcomes beyond the initial hospitalization.

## Conclusions

There was an increase in the number of patients with a mental health or substance use disorder comorbidity presenting to our hospital from the pre-pandemic to post-pandemic periods. The patients presenting during the pandemic period had the highest ISS, ICU days, and utilized the most operative resources, indicating a higher injury severity during this time. Further research should explore whether these trends represent temporary pandemic effects or signal a fundamental shift requiring sustained adaptation of pediatric trauma care systems to adequately care for this vulnerable population.

These findings should be considered when developing targeted prevention strategies for patients with mental health and substance abuse disorders, particularly during and after public health emergencies. Enhanced mental health screening in emergency and trauma settings, partnered with prompt and efficient referral systems for appropriate follow-up care, may help address the needs of the pediatric patient population.
